# Effectiveness of training programs based on mindfulness in reducing psychological distress and promoting well-being in medical students: a systematic review and meta-analysis

**DOI:** 10.1186/s13643-023-02244-y

**Published:** 2023-05-05

**Authors:** Claudia Cardoso Gomes da Silva, Cláudia Vicari Bolognani, Fábio Ferreira Amorim, Aline Mizusaki Imoto

**Affiliations:** 1 Programa de Pós-Graduação em Ciências da Saúde, Coordenação de Cursos Pós-Graduação Stricto Sensu, Escola Superior de Ciências da Saúde (ESCS), Setor Médico Hospitalar Norte, Conjunto A, Bloco 01, Edifício Fepecs - Asa Norte, Brasília, DF 70710-907 Brazil; 2 Coordenação de Pesquisa e Comunicação Científica, Escola Superior de Ciências da Saúde (ESCS), Setor Médico Hospitalar Norte, Conjunto A, Bloco 01, Edifício Fepecs - Asa Norte, Brasília, DF 70710-907 Brazil

**Keywords:** Medical students, Undergraduate medical students, Medical education, Mindfulness, Stress management, Psychological health, Mental health, Mindfulness-based stress reduction, Systematic review, Meta-analysis

## Abstract

**Background:**

Medical schools have used mindfulness meditation as a strategy to assist students in stress management. This study aimed to seek evidence regarding the effectiveness of mindfulness-based training programs in reducing psychological distress and promoting the well-being of medical students.

**Methods:**

We conducted a systematic review and meta-analysis. Cochrane Library, Embase, PubMed/MEDLINE, PsycINFO/PsycNet, LILACS/BVS, ERIC (ProQuest), Web of Science, OpenGrey, and Google Scholar were searched for randomized clinical trials published until March 2022, without time or language restrictions. Two authors independently screened the articles, extracted data using a standardized extraction form, and assessed the methodological quality of the included studies using the Cochrane’s Risk of Bias 2 (ROB 2) tool and the quality of evidence using the Grading of Recommendations Assessment, Development, and Evaluation (GRADE) tool.

**Results:**

Of the 848 articles retrieved, 8 met the inclusion criteria. Mindfulness-based training improved the outcomes: mindfulness (small post-intervention effect: SMD = 0.29; 95% CI: 0.03 to 0.54; *p* = 0.03; *I*^2^ = 46%; high evidence quality, and small effect at follow-up: SMD = 0.37; 95% CI: 0.04 to 0.70; *p* = 0.03; *I*^2^ = 53%; low evidence quality), psychological well-being/health (there was no statistically significant difference between the groups in the post-intervention effect: SMD =  − 0.27; 95% CI: − 0.67 to 0.13; *p* = 0.18; *I*^2^ = 76%; moderate evidence quality, and a significant difference at follow-up: SMD =  − 0.73; 95% CI: − 1.23 to − 0.23; *p* = 0.004; *I*^2^ = 61%; low evidence quality), and stress (small post-intervention effect: SMD =  − 0.29; CI of 95%: − 0.56 to − 0.02; *p* = 0.04; *I*^2^ = 57%; moderate evidence quality, and moderate effect at follow-up: SMD =  − 0.45, 95% CI: − 0.67 to − 0.22, *p* = 0.0001, *I*^2^ = 0%, moderate evidence quality). The quality of evidence for the anxiety, depression, and resilience outcomes is low and for the empathy outcome, very low.

**Conclusion:**

The results indicate that the students who participated in the mindfulness training perceived improvements in the stress and psychological distress symptoms and improved health perception and psychological well-being. However, the significant heterogeneity among studies should be considered when interpreting these findings.

**Systematic review registration:**

PROSPERO CRD42020153169.

## Background

Medical students are affected by several stress-generating factors. Among the most cited in the literature, we find a demanding study load, leading to physical and mental exhaustion; high competitiveness among peers; poor quality of life, including sleep deprivation and irregular eating; pressures and abuses of power by professors; high levels of self-demand; social isolation; and no time for leisure [1–7].

Immersion in scenarios of suffering, pain, serious illnesses, and death also reinforces the need for future physicians to develop skills to take care of their health and quality of life [1, 7]. Studies show that about a fifth of university students worldwide develop some type of psychiatric disorder during their academic life, with rates higher than those presented by non-university students and the general population [8–10]. Among the mental disorders observed in this population, depression and anxiety disorders are the most frequent [11–16].

Considering this context of psychological distress, medical schools are responsible for ensuring the health of their students, offering prevention, care strategies, and conditions for the full development of future physicians so that they can reach the expected profile to exercise their role in society [2, 3, 5, 14, 16–22]. Among the strategies to promote the well-being of the university population, the most cited by medical schools in different countries worldwide are meditation techniques that use training models known as mindfulness training [1, 6, 23–26].

Mindfulness is a state of awareness that emerges by paying attention, on purpose, in the present moment, without judging the experience that happens moment by moment [27]. The technique includes the regular practice of formal meditation and informal practices designed to expand awareness into all aspects of life [28]. Galante et al. [25] present mindfulness training as an option among mental health promotion strategies for university students. Adherence to attention regulation training would be perceived as the acquisition of an ability to deal with stressful situations, such as the exam period, instead of using a conventional health intervention.

University of Massachusetts Medical School, USA, was the first medical school to offer a mindfulness-based stress reduction (MBSR) program as a part of its curriculum, in 1985 [29]. Barnes et al. [30] show that, in 2014, almost two-thirds of medical schools in the USA already offered mindfulness-based programs as an option for self-care and psychological distress management.

Mindfulness meditation has its origins in the Buddhist tradition but has been adapted to the Western context for therapeutic use, regardless of its religious aspect. This type of meditation is the essence of a stress reduction program that Jon Kabat-Zinn started developing at the University of Massachusetts in 1979, known as MBSR or Stress Reduction Program (SRP) [31, 32]. The MBSR was initially developed within a university hospital to manage chronic pain and is now widely used to reduce psychological morbidity associated with chronic illnesses and treat emotional and behavioral disorders [32]. The technique influences several cognitive functions, including attention, perception, self-regulation, self-monitoring, memory, planning, decision-making, logic, and inhibitory control. In addition, meditation makes the brain areas associated with happiness, empathy, and compassion more active [33].

According to Chen et al. [34], mindfulness is an emerging concept in the health field, confirmed as an effective tool to help individuals control emotional and clinical symptoms. By modifying brain activity, meditation practice causes changes in brain areas responsible for cognitive and emotional functions [21, 25, 33]. The study by Hölzel et al. [35] suggests that mindfulness meditation would be associated with neuroplastic changes in the anterior cingulate cortex, insula, temporo-parietal junction, fronto-limbic network, and network structures, which would act synergistically, establishing a process of enhanced self-regulation. This mechanism would be responsible for the psychological change and physical and mental well-being, verified by the following effects: increased attention, increased body awareness, emotion regulation, metacognitive development, and change of perspective on self.

Mindfulness meditation is related to qualities of attention and awareness. Therefore, it could be defined, within the psychological context, as a mental state that is characterized by self-regulation of attention to the present experience, in an open, curious, broad, and tolerant attitude, directed towards all phenomena that manifest themselves in the conscious mind, that is, all kinds of thoughts, fantasies, memories, sensations, and emotions experienced in the field of attention are perceived and accepted as they are [31, 32, 36].

According to Tang et al. [37], the research fields of Psychology and Neuroscience have increased the number of studies on mindfulness meditation, evidencing that its practice has collaborated to the reduction of stress and the promotion of health, with developments in cognitive performance and the physical and mental health of its practitioners. Regarding the positive effects of mindfulness meditation on psychological health, studies have reported increased subjective well-being, reduction of psychiatric and stress-related symptoms, decreased emotional reactivity, and improved behavioral regulation [33, 35, 37–40].

Given the growing number of publications on mindfulness-based training offered to medical students as an option for psychological distress prevention, the proposition of a systematic review exclusively including randomized clinical trials to understand whether the MBSR program is a suitable tool to be used by medical schools is justified. This study aimed to assess the scientific evidence available on the effectiveness of mindfulness-based training programs offered by medical schools in improving the psychological states of mindfulness, well-being, stress, anxiety, depression, resilience, and empathy of medical students.

## Methods

A systematic review and meta-analysis were conducted on evidence regarding the effectiveness of mindfulness-based training programs in reducing psychological distress and promoting the well-being of medical students. The protocol was registered in the International Prospective Registry of Systematic Reviews (PROSPERO) under number CRD42020153169. The methodology was based on the Cochrane Collaboration Handbook [41] and was reported following the Preferred Reporting Items for Systematic Reviews and Meta-Analyses (PRISMA) guidelines [42].

### Eligibility criteria

A systematic search was carried out for peer-reviewed manuscripts published until March 2022, without initial time or language restrictions. We used the population, intervention, comparison, outcome, and study design (PICOS) framework to guide our study selection: P (population) = medical students; I (intervention) = mindfulness meditation training; C (comparison) = control group without intervention, waiting list, or different classroom; O (outcome) = psychological distress, well-being; and S (study design) = randomized clinical trial.

### Population

Studies that include medical students over 18 years old, with or without diagnosed anxiety and depression.

### Intervention

This review included only studies evaluating mindfulness training based on the original MBSR program by Kabat-Zinn [31] offered to medical students and targeted at the academic, personal, and professional aspects of their lives. The original program is mainly characterized by teaching different meditation techniques (seated meditation, body sweeping, yoga movements, and walking meditation) at weekly meetings over 8 weeks, including mindfulness exercises for daily activities and practice at home [27, 31, 36]. Both face-to-face training and distance learning courses, such as by online courses, compact discs (CD), and cell phone applications, were included.

### Control

We only included studies with a control or comparison group, such as a non-intervention group and persons on the waiting list for a mindfulness-based program or in a classroom where the program was not offered. Studies without a control or comparison condition were excluded.

### Outcomes

Primary outcomes were psychological state of mindfulness, well-being, stress, anxiety, and depression. Secondary outcomes were resilience and empathy. All outcomes were assessed using self-report questionnaires validated according to each outcome and language.

### Study design

We only included randomized clinical trials that assessed the effectiveness of mindfulness-based training programs in reducing psychological distress and promoting the well-being of medical students.

### Search strategy

Cochrane Library, Embase, PubMed/MEDLINE, PsycINFO/PsycNet, LILACS/BVS, ERIC (ProQuest), Web of Science, OpenGrey, and Google Scholar were searched for randomized clinical trials published until March 15, 2022, without time or language restrictions. The following search terms were used as reference: “medical students;” “students, medical;” “medical education;” “mindfulness;” “mindfulness meditation;” “zen;” “vipassana;” “mindfulness-based stress reduction;” “MBSR;” and “Mind–body skill training.” The search strategies are detailed in Table 1.

**Table 1:** Electronic search strategies

Cochrane Library 504 studies	#1 MeSH descriptor: [Students, Medical] explode all trees #2 (Medical Students) #3 (Student, Medical) #4 (Medical Student) #5 (Undergraduate medical students) #6 (medical education) #7 #1 or #2 or #3 or #4 or #5 or #6 #8 MeSH descriptor: [Mindfulness] explode all trees #9 (Mindfulness meditation) #10 (Zen) #11 (Vipassana) #12 (mindfulness-based stress reduction) #13 (mind–body skill training) #14 (MBSR) #15 #8 or #9 or #10 or #11 or #12 or #13 or #14 #16 #7 AND #15
Embase 666 studies	('medical student'/exp OR 'medical student' OR 'medical students'/exp OR 'medical students' OR 'undergraduate medical education'/exp OR 'undergraduate medical education' OR 'medical education'/exp OR 'medical education') AND ('mindfulness'/exp OR mindfulness OR 'mindfulness based stress reduction'/exp OR 'mindfulness based stress reduction' OR 'mindfulness training'/exp OR 'mindfulness training' OR 'mindfulness meditation'/exp OR 'mindfulness meditation' OR 'mindfulness based stress reduction program'/exp OR 'mindfulness based stress reduction program')
PubMed/MEDLINE 856 studies	("Students, Medical"[Mesh] or (Medical Students) or (Student, Medical) or (Medical Student) or (Undergraduate medical students) or (medical education)) AND ("Mindfulness"[Mesh] or (Mindfulness meditation) or (Zen) or (Vipassana) or (mindfulness-based stress reduction) or (mind–body skill training) or (MBSR))
PsycINFO/PsycNet 61 studies	((Students, Medical) or (Medical Students) or (Student, Medical) or (Medical Student) or (Undergraduate medical students) or (medical education) AND (Mindfulness) or (Mindfulness meditation) or (Zen) or (Vipassana) or (mindfulness-based stress reduction) or (mind–body skill training) or (MBSR))
LILACS/BVS 125 studies	(tw:("Estudantes de Medicina" or (MH:M01.848.769.602$))) AND (tw:("Atenção Plena" or (Consciência Plena) or (Mindfulness) or (MH:F02.463.551$) or (MH:F04.754.137.350.500$)))
ERIC (ProQuest) 92 studies	((Students, Medical) or (Medical Students) or (Student, Medical) or (Medical Student) or (Undergraduate medical students) or (medical education) AND (Mindfulness) or (Mindfulness meditation) or (Zen) or (Vipassana) or (mindfulness-based stress reduction) or (mind–body skill training) or (MBSR))
Web of Science 02 studies	((Students, Medical) or (Medical Students) or (Student, Medical) or (Medical Student) or (Undergraduate medical students) or (medical education) AND (Mindfulness) or (Mindfulness meditation) or (Zen) or (Vipassana) or (mindfulness-based stress reduction) or (mind–body skill training) or (MBSR))
OpenGrey 0 studies	students, medical or Undergraduate medical students or medical education and mindfulness or Mindfulness meditation or Zen or Vipassana or mindfulness-based stress reduction or mind–body skill training
Google Scholar 179 studies	students, medical or Undergraduate medical students or medical education and mindfulness or Mindfulness meditation or Zen or Vipassana or mindfulness-based stress reduction or mind–body skill training

### Study selection

**Fig. 1:**
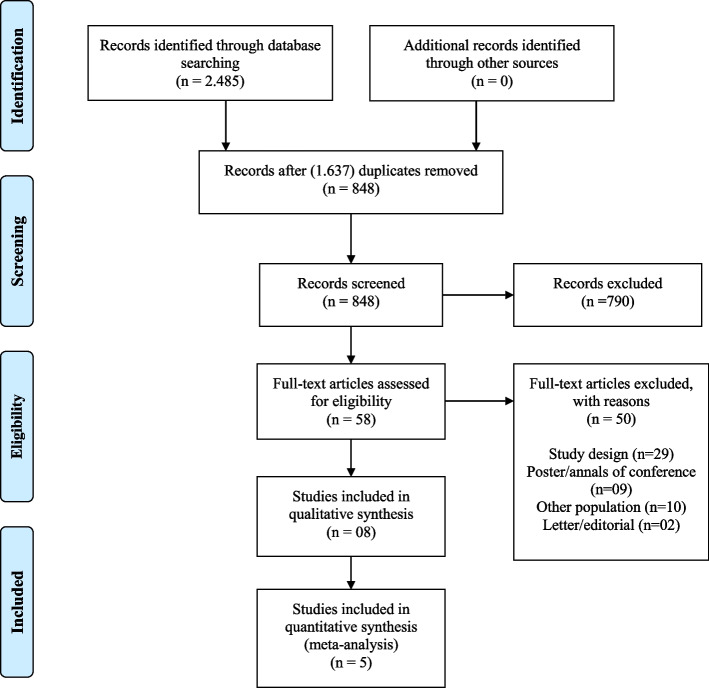
PRISMA flow diagram: study selection process

The titles and abstracts identified through all search sources were downloaded to EndNote Basic® online (https://endnote.com), where duplicates were removed. Then, the studies were uploaded to Covidence (https://www.covidence.org), as recommended by the Cochrane Collaboration, and screened using the eligibility criteria described above. Titles and abstracts were screened by two reviewers independently. The reviewers discussed disagreements about including or excluding a particular study until they reached a consensus or consulted with a third reviewer if required. After this stage, all full-text articles were assessed for relevance by the same two reviewers independently, who determined the final studies eligible for inclusion in the systematic review. Again, disagreements were resolved by mutual consensus or in consultation with the third reviewer, if required. Studies that did not meet the criteria were excluded, and related reasons were recorded. The study selection process was carried out according to the PRISMA Flow Diagram (http://www.equator-network.org/reporting-guidelines/prisma/), as shown in Fig. 1.

### Data extraction and management

Two reviewers independently extracted data using a standardized tool developed based on the Cochrane Collaboration recommendations, with further discussion of differences and consensus. Discrepancies in the extracted data were resolved through the involvement of a third reviewer if required.

The data collection followed the criteria of the study protocol addressing general study characteristics (author, year, title, journal, country and language, funding source, study design, public or private medical school); information about the participants (age, sex, ethnicity, diagnosis or specific characteristics, sample size); data on the intervention (technique description, duration of the intervention, session duration, mindfulness-based program attendance/compliance by the medical students, inclusion of a component of practice at home, target group or universal offer, mindfulness-training given by the school or by external specialists); control data (other types of intervention or control group condition); and, finally, outcome data (measurement methods, self-report or third-party assessment, time points for evaluation and post-intervention from 6-month to 1-year follow-up, evaluation of the training from the point of view of medical students, study conclusion).

### Risk of bias assessment

Two reviewers independently assessed the risk of bias using the Cochrane Risk of Bias 2 (RoB 2) tool 2019 version for the following outcomes at the latest follow-up [43]: Anxiety, Depression, Mindfulness, Well-being/Psychological Health, Stress, Empathy, and Resilience.

For all outcomes, the intervention assignment (ITT) was the effect of interest. Differences were resolved by a third reviewer if required. According to Sterne et al. [44], the five domains for assessing the risk of bias in randomized controlled trials are (1) bias arising from the randomization process, (2) bias due to deviations from intended interventions, (3) bias due to missing outcome data, (4) bias in measurement of the outcome, and (5) bias in selection of the reported result. The risk of bias assessments was managed using the RoB 2 Excel tool (https://methods.cochrane.org/risk-bias-2).

### Quality of the evidence: Cochrane GRADE assessment

The quality of evidence assessment refers to measuring the degree of confidence of an estimated effect of the intervention [45]. The instrument used for this evaluation was the Grading of Recommendations Assessment, Development and Evaluation (GRADE), a system developed to grade the quality of evidence and its strength for health recommendations [45]. This evaluation was processed for each outcome analyzed, obtaining the levels of evidence classification: (a) high (there is strong confidence that the true effect is close to the estimated one); (b) moderate (moderate confidence in the estimated effect); (c) low (the confidence in the effect is limited); and (d) very low (the confidence in the estimated effect is very limited, with an important degree of uncertainty in the findings) [45].

There is a set of factors used as a reference to raise or lower the quality of evidence from studies: study design, methodological limitations (risk of bias), inconsistency (heterogeneity), indirect evidence, imprecision (wide confidence interval), publication bias, the magnitude of effect, and residual confounding factors [45]. Two reviewers independently performed the assessment, discussing the differences subsequently. Discrepancies were resolved through the involvement of a third reviewer if required.

### Heterogeneity investigation

#### Assessment of statistical heterogeneity

The data heterogeneity was examined using the Higgins inconsistency test (*I*^2^), which describes the variability percentage of the effect estimate attributed to heterogeneity. An *I*^2^ value between 0 and 25% indicates mild (acceptable) heterogeneity; 25 to 50% moderate heterogeneity; and higher than 50% high heterogeneity [45].

#### Subgroup analysis and investigation of heterogeneity

Because mindfulness is an intervention with several characteristics such as duration, form of application, and different school contexts (curriculum, course phase in which the training, and student engagement in practice were applied), the possibility of clinical heterogeneity is relevant. The difference in the effect of the intervention may lead to statistical heterogeneity. Therefore, confidence intervals for individual study results (represented graphically by the horizontal lines) that had little overlap probably indicate the presence of statistical heterogeneity (variability in the intervention effects being evaluated in the different studies). In results that showed substantial heterogeneity, we investigated the possible reasons for this using subgroup analysis [41, 45]. We attempted to perform a subgroup analysis; however, due to the data heterogeneity, there was not enough data for this.

#### Sensitivity analyses

Sensitivity analyses are alternative analyses to assess the influence of each of the studies on the outcome. One way to investigate is to replicate the meta-analysis excluding in each stage one of the studies included in the review. This process can be repeated by eliminating one or more studies (for example, those with greater variability in the effect of the intervention) to determine their possible influence on the results. Results similar in direction, the magnitude of effect, and statistical significance indicate robust review findings [41, 45].

After heterogeneity testing, we investigated possible reasons for this using sensitivity analysis in the outcomes with substantial heterogeneity.

### Data synthesis

The results immediately after intervention and upon follow-up (from 6 month to 1 year) were extracted from the included studies, being collected through continuous data (mean and standard deviation) and the total number of participants. When numerical data were missing in a study, we contacted the authors requesting additional data. A meta-analysis was performed using the Review Manager Analysis software, version 5.3 (Cochrane Collaboration). Statistical significance was defined as *p* ≤ 0.05. Considering that the outcomes of interest were evaluated with different scales and units, standardized measurements were used to calculate the intervention effect sizes in standardized mean difference (SMD) and 95% confidence intervals (95% CI). A SMD below 0.4 indicates small effect; between 0.4 and 0.7, moderate effect; and above 0.7, large effect [41, 43]. The random-effects model was used for the meta-analysis.

## Results

Figure 1 summarizes the article selection process. After the search through the selected databases, 2485 studies were found, and the EndNote Basic® online platform automatically excluded 1637 duplicates. The remaining 848 studies were screened based on title and abstract. This process eliminated 790 studies, and 58 studies were submitted to a full-text read for eligibility. Then, 50 studies were excluded for the following reasons: (a) study design (29); (b) poster/annals of conference format (9); (c) other population (10), and (d) letter/editorial (2). After the screening, eight studies were selected for data extraction.

### Characteristics of the studies

Eight studies were included in this review. The main characteristics of these studies are demonstrated in Tables 2 and 3. Most of them were published in the last decade, six between 2011 and 2019 [46–51], and the other two [52, 53], by the end of the 90 s. Four studies are from the USA [47, 50, 52, 53]; one from Brazil [51]; one from the Netherlands [49]; one from Australia [46]; and one from Malaysia [48]. Despite such diverse countries, all selected articles were published in English. Five studies were performed in public universities and three in private universities. Half of the studies included declared to have received funding regarding the financial resources destined for this type of research. Considering the included articles, the total number of participants was 694. Age ranged from 18 to 25 years old, with 440 females and 254 males. As for the intervention performed, the students received mindfulness training based on the original MBSR program by Kabat-Zinn [31]. The control groups are mainly characterized by waiting lists [46–48, 50, 52, 53], and only two authors reported courses traditionally offered by the university (neurology clerkship [49] and a course on the organizational aspects of the medical school [51]). There was no uniformity concerning when and at what stage of their academic program the mindfulness training was offered to undergraduate medical students. Most authors chose the initial 1st to 3rd grades to offer this training [47, 48, 51, 53]. Two studies reported that the offer was associated with a specific discipline, Behavioral Medicine [52] and Neurology [49]; in another study, the training was offered to students from four different grades [50]; and only one study reported a training offer for the final grades [46].

**Table 2:** Summary of the main characteristics of the eight studies included in the review

	Warnecke et al. (2011) [46]	Erogul et al. (2014) [47]	Phang et al. (2015) [48]	van Dijk et al. (2017) [49]	Yang et al. (2018) [50]	Damião Neto et al. (2020) [51]	Astin (1997) [52]	Shapiro et al. (1998) [53]
**Country**								
Australia	✓							
Brazil						✓		
Malaysia			✓					
Netherlands				✓				
USA		✓			✓		✓	✓
**Setting**								
Public university		✓	✓			✓	✓	✓
Private university	✓			✓	✓			
**Students’ year on the medical school**								
First		✓	✓		✓	✓		✓
Second			✓		✓			✓
Third			✓		✓			
Fourth					✓			
Fifth	✓							
Sixth	✓							
Not specified				✓			✓	
**Length of the MBSR program**								
4-weeks					✓			
5-weeks			✓					
6-weeks						✓		
7-weeks								✓
8-weeks	✓	✓		✓			✓	
**Characteristic of the MBSR program**								
Self-guided course	✓				✓			
Face-to-face meetings and home practice		✓	✓	✓		✓	✓	✓
**Outcomes**								
Mindfulness			✓	✓	✓	✓		
Well-being/Psychological Health		✓		✓	✓	✓		
Stress	✓	✓	✓	✓	✓	✓		
Anxiety	✓					✓	✓	✓
Depression	✓					✓	✓	✓
Empathy				✓				✓
Resilience		✓						
**Follow-up**								
At the end of the intervention	✓	✓	✓	✓	✓	✓	✓	✓
6 months to 1 year	✓	✓	✓	✓

**Table 3:** General characteristics of the included studies

Study ID	Participants	Intervention	Comparator	Outcome	Time frame measurement	Notes
Warneck et al. (2011) [46] Australia	Medical students in the final 2 years of their degree course Location: distributed across three clinical schools attached to the University of Tasmania, Hobart, Tasmania, Australia Number of students randomized = 65 Intervention group = 31 Control group = 34	8-week mindfulness-based intervention program (adapted from Kabat-Zinn, 1982), 28 h of practice In addition: - 30 min audio, CD of guided mindfulness practice, followed independently every day - Adherence diary - No face-to-face meetings nor interaction between the instructors and students	The mindfulness intervention CD after the 8-week trial period as an incentive to remain in the trial	- Perception of stress: PSS - Depression, Anxiety and Stress: DASS	T1: before intervention T2: following the intervention (8 weeks) T3: 16 weeks (4 months) following the intervention	Funding source: the Australian and New Zealand Association for Health Professional Educators (ANZAHPE) Declarations of interest: no conflicts
Erogul, et al. (2014) [47] USA	First-year class of students Location: SUNY Downstate School of Medicine in Brooklyn, New York, USA Number of students randomized = 57 Intervention group = 28 Control group = 29	8-week mindfulness-based intervention program Group (adapted from Kabat-Zinn, 1982), 10 h of practice In addition: - Between the 7th and 8th weekly meeting, full-day retreat - Home practice: individual sessions of daily meditation for 20 min for the 8-week intervention	The control group did not receive any intervention during the 8-week study period	- Perceived stress: PSS - Resilience: RS	T1: before the intervention T2: after the intervention T3: 6 months after the intervention	Funding source: Arnold P. Gold Foundation Declarations of interest: none reported
Phang et al. (2015) [48] Malaysia	Medical students in year 1 to 3 of studies in Universiti Putra Malaysia (UPM), Malaysia Number of students randomized = 75 Intervention group = 37 Control group = 38	“Mindful-Gym”: 5-week mindfulness-based intervention program Group (adapted from Kabat-Zinn, 1982), 10 h of practice In addition: - Shorter in duration with more emphasis on informal practice, includes sessions on gratitude and cultivation of loving-kindness, and contains instructions tailored for medical students - Home practice: daily self-help exercises audio-guided instructions on a CD	They were informed that they would receive the program in a DVD 6 months later (after the follow-up period)	- Level of awareness and mindful attention: MAAS - Perceived stress: PSS - Mental distress: GHQ	T1: 1 week before intervention T2: 1 week after the interventions T3: 6 months after the interventions	Funding source: The Department of Psychiatry, Faculty of Medicine & Health Sciences, UPM, and a research grant from UPM Declarations of interest: none reported
van Dijk et al. (2017) [49]	Medical students in first-year clinical neurology clerkship, in Radboudumc University in Nijmegen, Netherlands Number of students randomized = 167 Intervention group = 83 Control group = 84	8-week mindfulness-based intervention program Group (adapted from Kabat-Zinn, 1982), 16 h of practice In addition: - Addition of 10 min of interactive presentation each week related to the session - No silent retreat - Adaptation of the folder material for use in clinical clerkship students instead of patients - Home practice: online questionnaire that asked how much time, on average, they had spent each week on home practice	Clinical clerkships as usual in first-year clinical clerkship students	- Psychological distress: BSI - Positive mental health: MHC-SF - Physician empathy: JSPE - Mindfulness skills: FFMQ	T1: before intervention T2: 3 months after T1 T3: 7 months after T1 T4: 12 months afterT1 T5: 15 months after T1 T6: 20 months after T1	Funding source: The Department of Psychiatry and Department of Primary and Community Care of the Radboudumc and by a grant of the Department of Evaluation, Quality and Development of Education of the Radboudumc Declarations of interest: none reported
Yang et al. (2018) [50] USA	Medical students at Keck School of Medicine at the University of Southern California (USC), USA Number of students randomized = 88 Intervention group = 45 Control group = 43	4-week mindfulness-based intervention program (adapted from Kabat-Zinn, 1982), 7.08 h of practice **In addition:** - Smartphone application "Headspace", an audio-guided mindfulness meditation program, structured such that each session lasts 10 min for the first ten days, 15 min for the next 15 days, and 20 min for all subsequent sessions - No face-to-face meetings nor interaction between the instructors and students	Waiting list	- Stress: PSS - Mindfulness: FFMQ - Well-being: GWBS	T1: before intervention T2: 30-day (1 month) time point T3: 60-day (2 months) time point	Funding source: none reported Declarations of interest: none reported
Damião Neto et al. (2020) [51] Brazil	First-year incoming medical students at the School of Medicine, Federal University of Juiz de Fora (UFJF), Juiz de Fora – Minas Gerais – Brazil Number of students randomized = 141 Intervention group = 70 Control group = 71	6-week mindfulness-based intervention program Group (adapted from Kabat-Zinn, 1982), 12 h of practice In addition: - Students stayed in a conventional classroom with school chairs - Home practice: four audios in MP3 format via email for guided home practice and practice daily for at least 10 min and bring a meditation diary including information of how many days students had practiced at home	The control group was given theoretical content in which they were shown organizational aspects of medical school	- Depression, Anxiety, and Stress: Depression, Anxiety, and Stress Scale (DASS) - Quality of Life: WHOQOL-BREF - Mindfulness: FFMQ-BR	T1: before intervention T2: after intervention	Funding source: none reported Declarations of interest: no conflict of interest
Astin (1997) [52] USA	Undergraduate students in an upper-division Behavioral Medicine class Location: University of California, Irvine, USA Number of students randomized = 28 Intervention group = 14 Control group = 14	8-week mindfulness-based intervention program group (adapted from Kabat-Zinn, 1982), 16 h of practice In addition: - No all-day meeting - Home practice (cassette tapes) for 45 min/ day, 5 days/ week	Waiting list	**-** Psychological distress: Hopkins SC-90-R and subscales	T1: before intervention T2: following the intervention (8-weeks) T3: 6–9 months following the intervention	Funding source: none reported Declarations of interest: none reported
Shapiro et al. (1998) [53] USA	Premedical and medical students Inclusion criteria: students willing to be randomly assigned to either the intervention Location: The University of Arizona College of Medicine – Tucson, USA Number of students randomized = 73 Intervention group = 36 Control group = 37	7-week mindfulness-based intervention program group (adapted from Kabat-Zinn, 1982), 17.5 h of practice In addition: a “loving-kindness” and a “forgiveness” meditation were introduced and a weekly home practice assignments as well as daily journals	waiting list	- Empathy: ECRS - Psychological distress: SCL-90-R - Depression:subscale 4 of the SCL-90 **-** State and trait Anxiety: STAI Form Y	T1: before intervention T2: following the intervention (7 weeks)	Funding source: none reported Declarations of interest: none reported

*CD* compact disk, *PSS* Perceived Stress Scale, *DASS* Depression, Anxiety and Stress Scale, *RS* Resilience Scale; *DVD* digital versatile disc, *MASS* Mindful Attention Awareness Scale, *GHQ* General Health Questionnaire, *BSI* Brief Symptom Inventory, *MHC-SF* Mental Health Continuum-Short Form, *JSPE* Jefferson Scale of Physician Empathy, *FFMQ* Five Facet Mindfulness Questionnaire, *FFMQ-BR* Five Facet Mindfulness Questionnaire, *GWBS* General Well-Being Schedule, *WHOQOL-BREF* World Health Organization Quality of Life, *ECRS* Empathy Construct Rating Scale, *SCL-90-R* The Hopkins Symptom Checklist 90 Revised, *STAI – Form Y* The State-Trait Anxiety Inventory- Form Y

Table 3 made in the original MBSR program is related to the total hours of training, the focus of group discussions, and reading materials, which had their language adapted to the audience of medical schools. Only one of the studies [47] held the full-day retreat for mindfulness practice included in the original MBSR program. Four studies maintained the total intervention duration of 8 weeks [46, 47, 49, 52], although they presented different total hours of training. The studies by Shapiro et al. [53] and Damião Neto et al. [51] performed interventions that lasted 7 and 6 weeks, respectively. Unlike the others, Phang et al. [48] opted for 5 weeks, while Yang et al. [50], for only 4 weeks. The mean training time was 14.5 h. The studies by Warnecke et al. [46] and Yang et al. [50] innovated with daily practice proposals guided by a CD audio and cell phone application, respectively, developed for the studies instead of face-to-face meetings. However, the other authors reported having encouraged students participating in their studies to perform the practices learned in face-to-face meetings at home, preferably daily.

Although the practice at home was part of all studies, not all authors reported the mean hours or days the students managed to practice during the training period. Astin [52] reported that students’ mean time of practice was 30 min a day, 3.5 days a week. Warnecke et al. [46] found that the mean number of days the students practiced at home was 26.7 days, considering a 56-day follow-up. Erogul et al. [47] found that students’ mean time of practice was 40.7 min throughout the program when the expected practice was 140 min. Phang et al. [48] used a Likert-type scale with a score from 1 to 5 to verify the at-home practice by students, reporting a mean score of 3, which represents 2.5 days approximately half of the scheduled days. van Dijk et al. [49] also used a Likert-type scale, but with a score from 0 to 5, reporting a mean score of 1, representing 1 to 15 min of practice at home per day.

As for the outcomes analyzed in the included studies, different instruments were used as assessment measures for the same constructs through self-report questionnaires, i.e., the students assessed themselves at all time points evaluated. It should be highlighted those outcomes were divided into categories, but all measurement instruments used directly or indirectly assessed the students’ mental/psychological health aspects listed in the present study, including mindfulness, well-being, stress, anxiety, depression, resilience, and empathy. To assess stress/psychological distress, the authors chose the following scales: The Hopkins Symptom Checklist 90 Revised (SCL-90-R/GSI) [52, 53], Perceived Stress Scale (PSS-10) [46–48, 50], General Health Questionnaire (GHQ) [48], Brief Symptom Inventory (BSI/ GSI) [49], and Depression, Anxiety, and Stress Scale (DASS) [46, 51]. To assess anxiety, the scales used were as follows: SCL-90-R [52, 53], The State-Trait Anxiety Inventory (STAI-1 Form) [53], and DASS [46, 51]. To assess depression, the scales used were the following: SCL-90-R/ GSI [52, 53] and DASS [46, 51]. The instruments used to measure stress, anxiety, and depression presented scores in which lower values indicate lower levels of the evaluated outcomes. The mindfulness (level of awareness and attention to the experience at the present moment), an outcome directly linked to the intervention, was estimated by the scores The Mindful Attention Awareness Scale (MAAS) [48] and Five Facet Mindfulness Questionnaire (FFMQ) [49–51]. Yang et al. [50] reported only the “observing” subscale results. The outcome of well-being/psychological health was verified using the scales Mental Health Continuum-Short Form (MHC-SF) [49], General Well-Being Schedule (GWBS) [50], and World Health Organization Quality of Life (WHOQOL-BREF) [51]. The students’ level of empathy was verified by Shapiro et al. [53] and van Dijk et al. [49] using the scales Empathy Construct Rating Scale (ECRS) and Jefferson Scale of Physician Empathy (JSPE), respectively. Resilience was measured only by Erogul et al. [47], through the Resilience Scale (RS-14) instrument. The instruments used to assess the mindfulness, well-being, empathy, and resilience levels presented scores. Higher values indicate higher levels of the evaluated outcomes, expressing a negative relationship with psychological symptoms related to depression, anxiety, and stress/psychological distress.

### Risk of bias assessment per outcomes

#### Anxiety and depression

In the outcomes anxiety and depression assessed at the end of the intervention, the risk of bias was considered as overall some concerns of bias as three of four of the studies [46, 51, 53] were at some concerns of bias in the domain selection of the reported result [46, 52, 53] because it is not known whether the analysis was performed according to a plan. In addition, the randomization process was also considered as some concerns in the study of Damião Neto et al. [51] and Astin [52] presented a high risk of bias in the randomization process (Tables 4 and 5).

**Table 4:** Detailed risk of bias judgment by domains for Anxiety (Rob2 Tool): at the end of the intervention

**Domains**	Astin [52]	Shapiro et al. [53]	Warnecke et al. [46]	Damião Neto et al. [51]
Domain 1. Randomization process	High	Low	Low	Some concerns
Domain 2. Deviations from intended interventions	Some concerns	Low	Low	Low
Domain 3. Missing outcome data	Some concerns	Low	Low	Low
Domain 4. Measurement of the outcome	Some concerns	Low	Low	Some concerns
Domain 5. Selection of the reported result	Some concerns	Some concerns	Some concerns	Low
Domain 6. Overall bias	High	Some concerns	Some concerns	Some concerns

**Table 5:** Detailed risk of bias judgment by domains for Depression (Rob2 Tool): at the end of the intervention

Domains	Astin [52]	Shapiro et al. [53]	Warnecke et al. [46]	Damião Neto et al. [51]
Domain 1. Randomization process	High	Low	Low	Some concerns
Domain 2. Deviations from intended interventions	Some concerns	Low	Low	Low
Domain 3. Missing outcome data	Some concerns	Low	Low	Low
Domain 4. Measurement of the outcome	Some concerns	Low	Low	Some concerns
Domain 5. Selection of the reported result	Some concerns	Some concerns	Some concerns	Low
Domain 6. Overall bias	High	Some concerns	Some concerns	Some concerns

Of the four studies, only Warnecke et al. [46] assessed such outcomes at follow-up. The risk of bias was considered as overall some concerns (Tables 6 and 7).

**Table 6:** Detailed risk of bias judgment by domains for Anxiety (Rob2 Tool): 6-month to 1-year follow-up

Domains	Warnecke et al. [46]
Domain 1. Randomization process	Low
Domain 2. Deviations from intended interventions	Low
Domain 3. Missing outcome data	Low
Domain 4. Measurement of the outcome	Low
Domain 5. Selection of the reported result	Some concerns
Domain 6. Overall bias	Some concerns

**Table 7:** Detailed risk of bias judgment by domains for Depression (Rob2 Tool): 6-month to 1-year follow-up

Domains	Warnecke et al. [46]
Domain 1. Randomization process	Low
Domain 2. Deviations from intended interventions	Low
Domain 3. Missing outcome data	Low
Domain 4. Measurement of the outcome	Low
Domain 5. Selection of the reported result	Some concerns
Domain 6. Overall bias	Some concerns

#### Mindfulness

Phang et al. [48], van Dijk et al. [49], Yang et al. [50], and Damião Neto et al. [51] assessed the outcome mindfulness at the end of the intervention. The assessment of the risk of bias was considered as some concerns in the measurement of the outcome domain [49–51] because of the possibility of the outcome assessment being influenced by the knowledge of the intervention received and the possibility of there having been selection of the reported result [48, 50] (Table 8).

**Table 8:** Detailed risk of bias judgment by domains for Mindfulness (Rob2 Tool): at the end of the intervention

**Domains**		Phang et al. [48]	van Dijk et al. [49]	Yang et al. [50]	Damião Neto et al. [51]
Domain 1. Randomization process	Judgment	Low	Low	Low	Some concerns
Domain 2. Deviations from intended interventions	Judgment	Low	Low	Low	Low
Domain 3. Missing outcome data	Judgment	Low	Low	Low	Low
Domain 4. Measurement of the outcome	Judgment	Low	Some concerns	Some concerns	Some concerns
Domain 5. Selection of the reported result	Judgment	Some concerns	Low	Some concerns	Low
Domain 6. Overall bias	Overall judgment	Low	Some concerns	Some concerns	Some concerns

Of the four studies, only Damião Neto et al. [51] did not assess this outcome at follow-up. The risk of bias was considered as overall some concerns in two of three studies [49, 50] and was considered low in the study of Phang et al. [48] (Table 9).

**Table 9:** Detailed risk of bias judgment by domains for Mindfulness (Rob2 Tool): 6-month to 1-year follow-up

**Domains**		Phang et al. [48]	van Dijk et al. [49]	Yang et al. [50]
Domain 1. Randomization process	Judgment	Low	Low	Low
Domain 2. Deviations from intended interventions	Judgment	Low	Low	Low
Domain 3. Missing outcome data	Judgment	Low	Low	Low
Domain 4. Measurement of the outcome	Judgment	Low	Some concerns	Some concerns
Domain 5. Selection of the reported result	Judgment	Some concerns	Low	Some concerns
Domain 6. Overall bias	Overall judgment	Low	Some concerns	Some concerns

#### Well-being/psychological health

Overall, at the end of the intervention, the risk of bias for the outcome well-being and psychological health was assessed by four studies [47, 49–51] as some concerns due to the possibility of selection of the reported result [47, 50] and influence of the evaluator in the assessment of the outcomes by knowledge of the intervention received (Table 10).

**Table 10:** Detailed risk of bias judgment by domains for Well-being/Psychological Health (Rob2 Tool): at the end of the intervention

Domains		Erogul et al. [47]	van Dijk et al. [49]	Yang et al. [50]	Damião Neto et al. [51]
Domain 1. Randomization process	Judgment	Low	Low	Low	Some concerns
Domain 2. Deviations from intended interventions	Judgment	Low	Low	Low	Low
Domain 3. Missing outcome data	Judgment	Low	Low	Low	Low
Domain 4. Measurement of the outcome	Judgment	Low	Some concerns	Some concerns	Some concerns
Domain 5. Selection of the reported result	Judgment	Some concerns	Low	Some concerns	Low
Domain 6. Overall bias	Overall judgment	Some concerns	Some concerns	Some concerns	Some concerns

At follow-up, two studies [47, 49] assessed the risk of bias for this outcome which was considered as overall some concerns (Table 11).

**Table 11:** Detailed risk of bias judgment by domains for Well-being/Psychological Health (Rob2 Tool): 6-month to 1-year follow-up

Domains		Erogul et al. [47]	van Dijk et al. [49]
Domain 1. Randomization process	Judgment	Low	Low
Domain 2. Deviations from intended interventions	Judgment	Low	Low
Domain 3. Missing outcome data	Judgment	Low	Low
Domain 4. Measurement of the outcome	Judgment	Low	Some concerns
Domain 5. Selection of the reported result	Judgment	Some concerns	Low
Domain 6. Overall bias	Overall judgment	Some concerns	Some concerns

#### Stress

Six studies [46–51] assessed the outcome stress at the end of the intervention. Overall, the assessment of the risk of bias was considered as some concerns, except in the study of Phang et al. [48]. Four of six studies [46–48, 50] were at some concerns of bias in the domain selection of the reported result, probably because it is not known whether the analysis was performed according to a plan. In addition, three studies presented some concerns in the domain measurement of the outcome due to the possibility of the outcome being influenced by the knowledge of the intervention received (Table 12).

**Table 12:** Detailed risk of bias judgment by domains for Stress (Rob2 Tool): at the end of the intervention

Domains		Warnecke et al. [46]	Erogul et al. [47]	Phang et al. [48]	van Dijk et al. [49]	Yang et al. [50]	Damião Neto et al. [51]
Domain 1. Randomization process	Judgment	Low	Low	Low	Low	Low	Some concerns
Domain 2. Deviations from intended interventions	Judgment	Low	Low	Low	Low	Low	Low
Domain 3. Missing outcome data	Judgment	Low	Low	Low	Low	Low	Low
Domain 4. Measurement of the outcome	Judgment	Low	Low	Low	Some concerns	Some concerns	Some concerns
Domain 5. Selection of the reported result	Judgment	Some concerns	Some concerns	Some concerns	Low	Some concerns	Low
Domain 6. Overall bias	Overall judgment	Some concerns	Some concerns	Low	Some concerns	Some concerns	Some concerns

At follow-up, four studies [46–49] assessed the risk of bias for this outcome which was considered as overall some concerns, except in the study of Phang et al. [48] (Table 13).

**Table 13:** Detailed risk of bias judgment by domains for Stress (Rob2 Tool): 6-month to 1-year follow-up

Domains		Warnecke et al. [46]	Erogul et al. [47]	Phang et al. [48]	van Dijk et al. [49]
Domain 1. Randomization process	Judgment	Low	Low	Low	Low
Domain 2. Deviations from intended interventions	Judgment	Low	Low	Low	Low
Domain 3. Missing outcome data	Judgment	Low	Low	Low	Low
Domain 4. Measurement of the outcome	Judgment	Low	Low	Low	Some concerns
Domain 5. Selection of the reported result	Judgment	Some concerns	Some concerns	Some concerns	Low
Domain 6. Overall bias	Overall judgment	Some concerns	Some concerns	Low	Some concerns

#### Empathy

Two studies [49, 53] assessed the outcome empathy at the end of the intervention. Overall, the assessment of the risk of bias was considered as some concerns because van Dijk et al. [49] presented some concerns of bias in the measurement of the outcome domain (possibility of the outcome being influenced by the knowledge of the intervention received), and Shapiro et al. [53] presented some concerns of bias in the selection of the reported result domain because it is not known whether the analysis was performed according to a plan (Table 14).

**Table 14:** Detailed risk of bias judgment by domains for Empathy (Rob2 Tool): at the end of the intervention

**Domains**		van Dijk et al. [49]	Shapiro et al. [53]
Domain 1. Randomization process	Judgment	Low	Low
Domain 2. Deviations from intended interventions	Judgment	Low	Low
Domain 3. Missing outcome data	Judgment	Low	Low
Domain 4. Measurement of the outcome	Judgment	Some concerns	Low
Domain 5. Selection of the reported result	Judgment	Low	Some concerns
Domain 6. Overall bias	Overall judgment	Some concerns	Some concerns

Only van Dijk et al. [49] assess this outcome at follow-up. The risk of bias was considered as overall some concerns (Table 15).

**Table 15:** Detailed risk of bias judgment by domains for Empathy (Rob2 Tool): 6-month to 1-year follow-up

Domains		van Dijk et al. [49]
Domain 1. Randomization process	Judgment	Low
Domain 2. Deviations from intended interventions	Judgment	Low
Domain 3. Missing outcome data	Judgment	Low
Domain 4. Measurement of the outcome	Judgment	Some concerns
Domain 5. Selection of the reported result	Judgment	Low
Domain 6. Overall bias	Overall judgment	Some concerns

#### Resilience

Erogul et al. [47] assessed the outcome resilience at the end of the intervention and 6-month to 1-year follow-up. The risk of bias was considered as overall some concerns due to the study presenting some concerns risk of bias in the selection of the reported result domain, so it is unknown whether the analysis was performed according to a plan (Table 16).

**Table 16:** Detailed risk of bias judgment by domains for Resilience (Rob2 Tool): at the end of the intervention and 6-month to 1-year follow-up

Domains		Erogul et al. [47]
Domain 1. Randomization process	Judgment	Low
Domain 2. Deviations from intended interventions	Judgment	Low
Domain 3. Missing outcome data	Judgment	Low
Domain 4. Measurement of the outcome	Judgment	Low
Domain 5. Selection of the reported result	Judgment	Some concerns
Domain 6. Overall bias	Overall judgment	Some concerns

To assess the size of the mindfulness training effect, our study analyzed the effects right after the end of the intervention and through a 6-month to 1-year follow-up. These effects were described through quantitative synthesis, where the outcomes resulted in sufficient data/studies for a meta-analysis (mindfulness, well-being/psychological health, and stress). For those outcomes in which it was not possible to carry out a meta-analysis (Anxiety, Depression, Empathy, and Resilience), the analyzable data from at least one primary study were used to calculate the intervention effect.

### Effects of mindfulness training on the mindfulness outcome

**Fig. 2:**
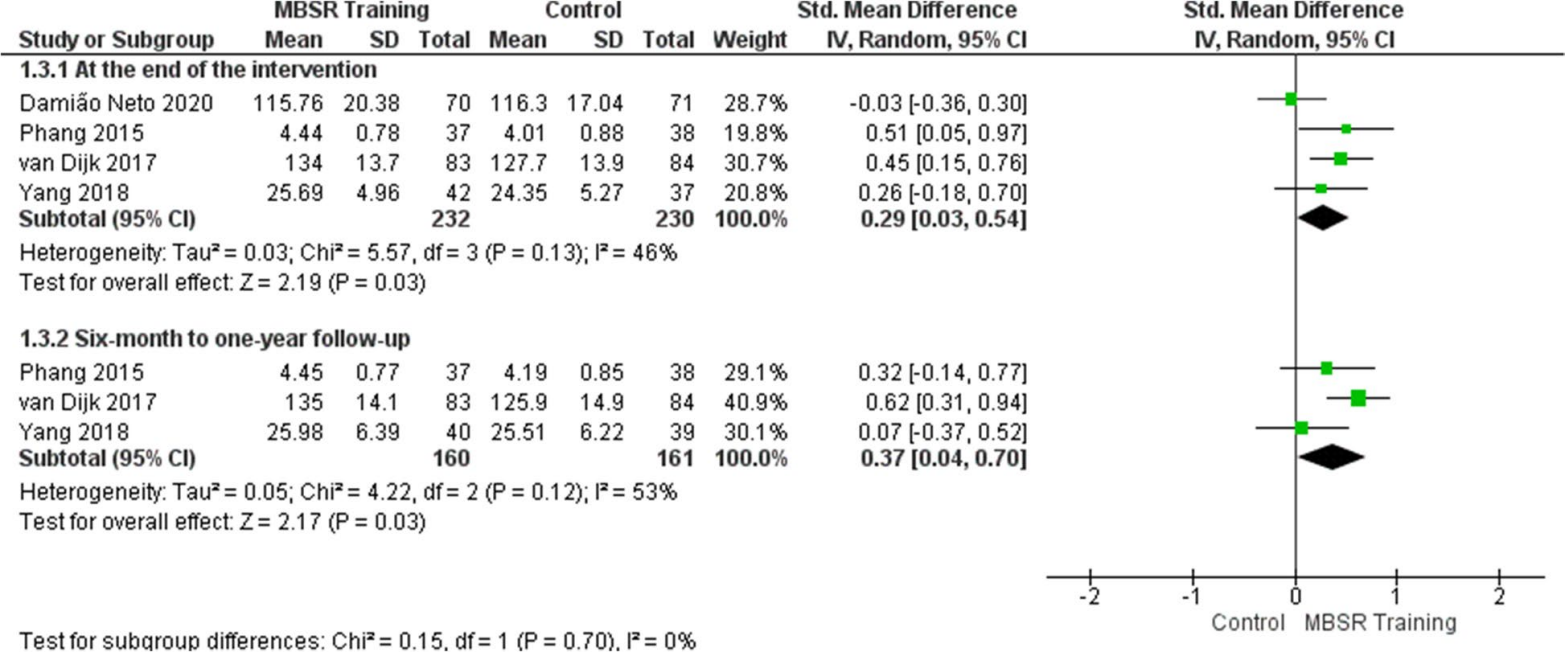
Effects of mindfulness training on the mindfulness outcome

At the end of the intervention, the meta-analysis of four studies [48–51] involving 462 students shows that mindfulness training improved the students’ mindfulness in the intervention group (Fig. 2). The effect is considered small (SMD = 0.29) and showed a statistically significant difference in the intervention group, with possible clinical relevance (SMD = 0.29; 95% CI: 0.03 to 0.54; *p* = 0.03; *I*^2^ = 46%). Heterogeneity among studies is moderate. Perhaps the heterogeneity could be explained by the articles that present different school contexts: curriculum, course phase in which the training, and student engagement in practice were applied. The study by Damião Neto et al. [51] showed less favorable results for the intervention when compared to the other studies, probably due to the moment of the medical course in which the training was offered (first year). Perhaps students do not identify the need to develop mindfulness skills as soon as they start medical school.

As for the 6-month to 1-year follow-up, the meta-analysis of three studies [48–50] involving 321 students shows mindfulness training increased the students’ mindfulness in the intervention group (Fig. 2). The effect is considered small (SMD = 0.37) and showed a statistically significant difference in the intervention group, with possible clinical relevance (SMD = 0.37; 95% CI: 0.04 to 0.70; *p* = 0.03; *I*^2^ = 53%). Heterogeneity among studies is high. In this case, the study by van Dijk et al. [49] presents more favorable results for the intervention when compared to the other studies. According to the authors, the students accepted the training well because it was integrated into the neurology internship, not characterized as an extra activity, and also because the format and content were adapted to the medical context. Perhaps this is because students in the clinical neurology internship, in addition to direct contact with patients, are confronted with a large volume of content to study, which may explain a greater appreciation of mindfulness skills and perception of the gains related to this learning.

The quality of evidence for the mindfulness outcome is high at the end of the intervention and low from 6-month to 1-year follow-up, as shown in the evidence summary (Table 17).

**Table 17:** Summary of evidence for this systematic review

Outcomes	Assessment time	Study	Effect		Studies (participants)	Certainty of evidence
			Mean score for control groups	Effect estimation		
Mindfulness	At the end of the intervention	Phang et al. (2015) [48] van Dijk et al. (2017) [49] Yang et al. (2018) [50] Damião Neto et al. (2020) [51]	-^a^	The mean scores for intervention groups were significantly higher than the mean scores for control groups (SMD: 0.29, 95% CI: 0.03 to 0.54)	4 (462)	⨁⨁⨁⨁ High
	6-month to 1-year follow-up	Phang et al. (2015) [48] van Dijk et al. (2017) [49] Yang et al. (2018) [50]	-^a^	The mean scores for intervention groups were significantly higher than the mean scores for control groups (SMD: 0.37, 95% CI: 0.04 to 0.7)	3 (321)	⨁⨁◯◯ Low ^b,i^
Well-being/Psychological Health	At the end of the intervention	Erogul et al. (2014) [47] van Dijk et al. (2017) [49] Yang et al. (2018) [50] Damião Neto et al. (2020) [51]	-^a^	The mean scores for intervention groups showed no significant difference compared to the mean scores for control groups (SMD: − 0.27, 95% CI: − 0.67 to 0.13)	4 (446)	⨁⨁⨁◯ Moderate^c^
	6-month to 1-year follow-up	Erogul et al. (2014) [47] van Dijk et al. (2017) [49]	-^a^	The mean scores for intervention groups were significantly lower than the mean scores for control groups (SMD: − 0.73, 95% CI: − 1.23 to − 0.23)	2 (224)	⨁⨁◯◯ Low^d,i^
Stress	At the end of the intervention	Erogul et al. (2014) [47] Phang et al. (2015) [48] van Dijk et al. (2017) [49] Yang et al. (2018) [50] Damião Neto et al. (2020) [51]	-^a^	The mean scores for intervention groups were significantly lower than the mean scores for control groups (SMD: − 0.29, 95% CI: − 0.56 to − 0.02)	5 (520)	⨁⨁⨁◯ Moderate^e^
	6-month to 1-year follow-up	Erogul et al. (2014) [47] Phang et al. (2015) [48] van Dijk et al. (2017) [49]	-^a^	The mean scores for intervention groups were significantly lower than the mean scores for control groups (SMD: − 0.45, 95% CI: − 0.67 to − 0.22)	3 (290)	⨁⨁⨁◯ Moderate^i^
Anxiety^f^	At the end of the intervention	Damião Neto et al. (2020) [51] Warnecke et al. (2011) [46] ^g^ Astin (1997) [52] ^g^ Shapiro et al. (1998) [53] ^g^	The mean score in control group was 3.57 ± 3.45 ^h^	The estimated mean difference between the two groups (MD: − 0.17, 95% CI: − 1.32 to 0.98) ^h^	4 (307)	⨁⨁◯◯ Low ^i^^, j^
Depression^f^	At the end of the intervention	Damião Neto et al. (2020) [51] Warnecke et al. (2011) [46] ^g^ Astin (1997) [52] ^g^ Shapiro et al. (1998) [53] ^g^	The mean score in control group was 3.97 ± 3.28 ^h^	The estimated mean difference between the two groups (MD: 0.06, 95% CI: − 1.04 to 1.16) ^h^	4 (307)	⨁⨁◯◯ Low ^i^^, j^
Empathy	At the end of the intervention	van Dijk et al. (2017) [49] Shapiro (1998) [53] ^g^	The mean score in control group was 108.4 ± 10.0 k	The estimated mean difference between the two groups (MD: − 3.5, 95% CI: − 6.51 to − 0.49) ^k^	2 (240)	⨁◯◯◯ Very low ^i^^, l^
	6-month to 1-year follow-up	van Dijk et al. (2017) [49]	The mean score in control group was 109.8 ± 8.6	The mean score for the intervention group showed no significant difference compared to the mean score for the control group (MD: − 1.10, 95% CI: − 4.20 to 2.00)	1 (167)	⨁◯◯◯ Low ^i^^, m^
Resilience	At the end of the intervention	Erogul et al. (2014) [47]	The mean score in control group was 77.1 ± 14.1	The mean score for the intervention group showed no significant difference compared to the mean score for the control group (MD: − 3.40, 95% CI: − 9.91 to 3.11)	1 (57)	⨁⨁◯◯ Low ^i^^, m^
	6-month to 1-year follow-up	Erogul et al. (2014) [47]	The mean score in control group was 77.3 ± 12.5	The mean score for the intervention group was significantly higher than the mean score for the control group (MD: − 5.10, 95% CI: − 11.08 to 0.88)	1 (57)	⨁⨁◯◯ Low ^i^^, m^

*95% CI* 95% confidence interval, *MD* mean difference, *SMD* standardized mean difference

^a^ The outcome was measured on a variety of scales

^b^*I*^2^: 53%—Substantial heterogeneity among trials (*I*^2^ equal or more than 50%, equal or less than 90%). Therefore, the certainty of the evidence was downgraded by one level (inconsistency)

^c^*I*^2^: 76%—Substantial heterogeneity among trials (*I*^2^ equal or more than 50%, equal or less than 90%). Therefore, the certainty of the evidence was downgraded by one level (inconsistency)

^d^*I*^2^: 61%—Substantial heterogeneity among trials (*I*^2^ equal or more than 50%, equal or less than 90%). Therefore, the certainty of the evidence was downgraded by one level (inconsistency)

^e^*I*^2^: 57%—Substantial heterogeneity among trials (*I*^2^ equal or more than 50%, equal or less than 90%). Therefore, the certainty of the evidence was downgraded by one level (inconsistency)

^f^There was no analyzable data on the outcome from 6-month to 1-year follow-up

^g^Not reported data (mean and standard deviation)

^h^The result is based on the Damião Neto et al. (2020) [51] that reported no significant difference. Warnecke et al. (2011) [46], Astin (1997) [52], and Shapiro et al. (1998) [53] reported improvement with intervention, but they did not show data (mean and standard deviation)

^i^The number of patients is less than 400 (< 400). Therefore, the certainty of the evidence was downgraded by one level (imprecision)

^j^There was a significant heterogeneity considering this study. The certainty of the evidence was downgraded by one level. (inconsistency)

^k^The result is based on van Dijk et al. (2017) [49] which reported no significant difference. Shapiro et al. (1998) [53] reported improvement with intervention, but they did not show data (mean and standard deviation)

^l^The result is based on just one study that showed data (mean and standard deviation). There was heterogeneity considering this study and another study that reported improvement with intervention but did not show data (mean and standard deviation. Therefore, the certainty of the evidence was downgraded by two levels (inconsistency)

^m^The confidence intervals cross the line of no effect, i.e., wide confidence intervals. Therefore, the certainty of the evidence was downgraded by one level (imprecision)

### Effects of mindfulness training on the well-being/psychological health outcome

**Fig. 3:**
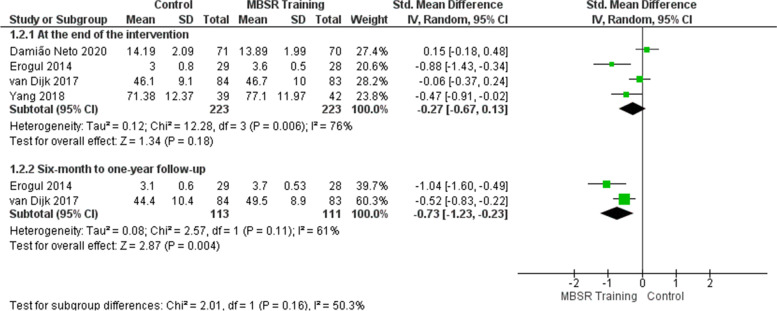
Effects of mindfulness training on the well-being/psychological health outcome

**Fig. 4:**
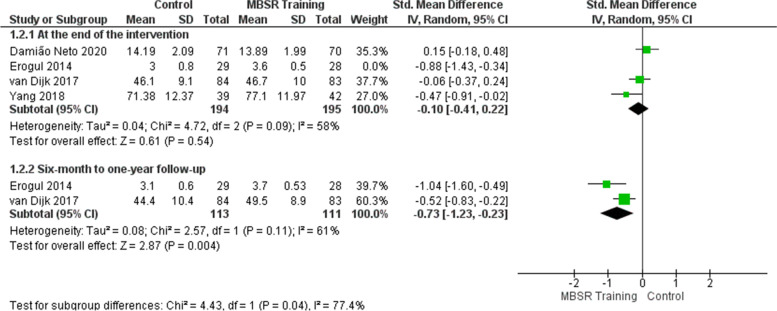
Sensitivity analysis: effects of mindfulness training on the well-being/psychological health outcome

At the end of the intervention, the meta-analysis of four studies [47, 49–51] did not show that mindfulness training improved the students’ perception of well-being/psychological health in the intervention group (Fig. 3). The effect showed no statistically significant difference between the control and intervention groups (SMD =  − 0.27; 95% CI: − 0.67 to 0.13; *p* = 0.18; *I*^2^ = 76%), although the perceived improvement of students who participated in the training represents a possible clinical relevance. Heterogeneity among studies is high. Sensitivity analysis was performed removing the study of Erogul et al. [47], which showed a discrepant result compared to the others. The heterogeneity decreased from 76 to 58% in the effect estimate (Fig. 4 and Table 18). To try to explain the more favorable outcome for the intervention in this study, we sought the authors’ explanations for the possible selection bias that occurred. Students from a first-year medical school class were randomized and divided into intervention and control groups. Still, consent for participation in the research was collected after randomization, so the authors recognized that students assigned to the intervention group may have accepted to participate because they had some preexisting allegiance to MBSR, which would also explain better results concerning the control group.

**Table 18:** Sensitivity analysis

Outcome	Heterogeneity before sensitivity analysis	Heterogeneity after sensitivity analysis	Study removed	Reason
Well-being/Psychological health	76%	58%	Erogul et al. [47]	The study showed a discrepant result compared to the others
Stress	57%	20%	Damião Neto et al. [51]	The study showed a discrepant result compared to the others

As for the 6-month to 1-year follow-up, the meta-analysis of two studies [47, 49] involving 224 students shows that mindfulness training improved the students’ perception of well-being/psychological health in the intervention group (Fig. 3). The effect is considered large (SMD =  − 0.73). There was a statistically significant difference in the intervention group, with possible clinical relevance (SMD =  − 0.73; 95% CI: − 1.23 to − 0.23; *p* = 0.004; *I*^2^ = 61%). Heterogeneity among studies is high. Perhaps the heterogeneity could be explained by the same reasons presented at the end of the intervention.

The quality of evidence for the well-being/psychological health outcome is moderate at the end of the intervention and low from 6-month to 1-year follow-up, as shown in the evidence summary (Table 17).

### Effects of mindfulness training on the stress outcome

**Fig. 5:**
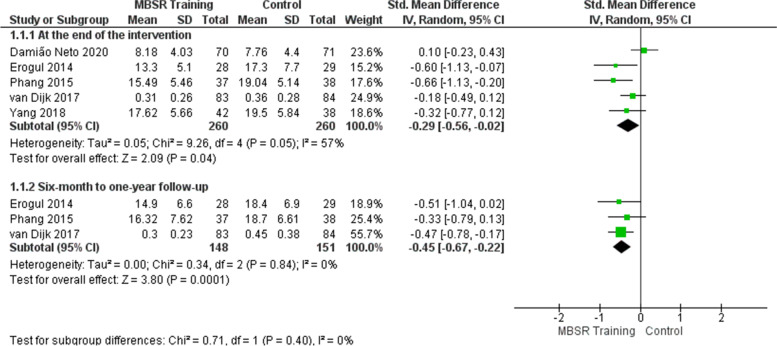
Effects of mindfulness training on the stress outcome

**Fig. 6:**
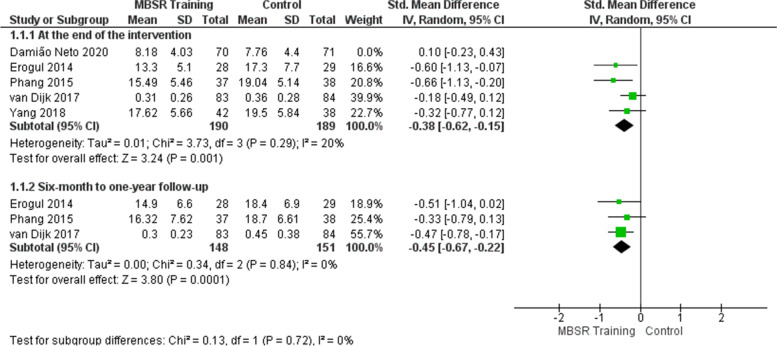
Sensitivity analysis: effects of mindfulness training on the stress outcome

At the end of the intervention, the meta-analysis of five studies [47–51] shows that mindfulness training reduced the students’ perception of stress/psychological distress in the intervention group (Fig. 5). The effect is considered small (SMD =  − 0.29) and showed a statistically significant difference in the intervention group, with possible clinical relevance (SMD =  − 0.29; 95% CI: − 0.56 to − 0.02; *p* = 0.04; *I*^2^ = 57%). Heterogeneity among studies is high. Sensitivity analysis was performed removing the study of Damião Neto et al. [51], which showed a discrepant result compared to the others. The heterogeneity decreased from 57 to 20% in the effect estimate (Fig. 6 and Table 18). One possible explanation is that the moment of application of the intervention was in the first year of the course, in which stress could not be developed. In addition, as it is a traditional teaching methodology, there was no contact with the patient (clinical subjects).

As for the 6-month to 1-year follow-up, the meta-analysis of three studies [47–49] involving 299 students shows that mindfulness training reduced the students’ perception of stress/psychological distress in the intervention group (Fig. 5). The effect is considered moderate (SMD =  − 0.45) and showed a statistically significant difference in the intervention group, with possible clinical relevance (SMD =  − 0.45; 95% CI: − 0.67 to − 0.22; *p* = 0.0001; *I*^2^ = 0%). Heterogeneity among studies is low.

The quality of evidence for the stress outcome is moderate at the end of the intervention and from 6-month to 1-year follow-up, as shown in the evidence summary (Table 17).

### Effects of mindfulness training on the anxiety outcome

Four studies [46, 51–53] involving 307 students used instruments to assess the anxiety outcome at the end of the intervention, but only Warnecke et al. [46] performed a 16-week follow-up after the intervention.

Astin [52], Shapiro et al. [53], and Warnecke et al. [46] did not present enough data to perform a meta-analysis for this outcome. Therefore, the only study with sufficient data to calculate the estimate of the intervention effect was the study by Damião Neto et al. [51], which did not find a significant difference between the scores measured before and after training or between the control and intervention groups (MD: − 0.17, 95% CI: − 1.32 to 0.98).

The quality of evidence for the anxiety outcome is low at the end of the intervention. There was no analyzable data on the outcome from 6-month to 1-year follow-up, as shown in the evidence summary (Table 17).

### Effects of mindfulness training on the depression outcome

Four studies [46, 51–53] involving 307 students used instruments to assess the depression outcome at the end of the intervention, but only Warnecke et al. [46] performed a 16-week follow-up after the intervention.

Astin [52], Shapiro et al. [53], and Warnecke et al. [46] did not present enough data to perform a meta-analysis for this outcome. Therefore, the only study with sufficient data to calculate the estimate of the intervention effect was the study by Damião Neto et al. [51], which did not find a significant difference between the scores measured before and after training or between the control and intervention groups (MD: 0.06, 95% CI: − 1.04 to 1.16).

The quality of evidence for the depression outcome is low at the end of the intervention. There was no analyzable data on the outcome from 6-month to 1-year follow-up, as shown in the evidence summary (Table 17).

### Effects of mindfulness training on the empathy outcome

Two studies [49, 53] used instruments to assess the empathy outcome at the end of the intervention, but only van Dijk et al. [49] performed a follow-up after the intervention.

Shapiro et al. [53] did not present enough data to perform a meta-analysis for this outcome. The study with sufficient data to calculate the estimate of the intervention effect was the study by van Dijk et al. [49], which found a significant improvement at the end of the intervention (MD: − 3.50; 95% CI: − 6.51 to − 0.49), but no significant effect until 80 weeks follow-up for this outcome (MD: − 1.10; 95% CI: − 4.20 to 2.00).

The quality of evidence for the empathy outcome is very low at the end of the intervention and low from 6-month to 1-year follow-up, as shown in the evidence summary (Table 17).

### Effects of mindfulness training on the resilience outcome

Erogul et al. [47] were the only ones to assess the students’ resilience after the intervention, showing no significant difference in the effect of the intervention at the end of the intervention (MD: − 3.40; 95% CI: − 9.91 to 3.11) and upon the 6-month follow-up (MD: − 5.10; 95% IC: − 11.08 to 0.88).

Despite this result, the study found a positive correlation between the resilience outcome and the stress and well-being perception outcomes, respectively, in both assessment time points.

There were not enough studies for a meta-analysis of this outcome.

The quality of evidence for the resilience outcome is low at the end of the intervention and from 6-month to 1-year follow-up, as shown in the evidence summary (Table 17).

## Discussion

This study aimed to assess whether mindfulness training programs adapted from the original MBSR program by Kabat-Zinn [31, 36] are effective in reducing psychological distress and promoting well-being, resilience, empathy, and mindfulness in medical students. The meta-analyses performed to assess the effect size of mindfulness training soon after the intervention revealed effects considered small for the mindfulness, well-being/psychological health, and stress outcomes. This result can be seen as a consequence of the heterogeneity among the analyzed studies, revealing a less accurate effect estimate due to the wider confidence intervals [45]. However, the fact that effects were considered small does not mean that the result has no clinical relevance. An aspect to be taken into consideration is that, although the intervention effect size is small, this population of students is not considered a clinical population but usually presents higher levels of psychological distress (stress, anxiety, and depression) in the initial assessments when compared to age-matched peers [1, 5, 54]. Among the studies included in the review, only one accepted students who already had a diagnosis of depression, referring them to the university’s mental health service for follow-up [48]. However, the other studies showed a certain degree of psychological distress in the initial assessments based on the students’ own perception of their emotional health. Warnecke et al. [46] confirmed that the initial assessment of medical students participating in their research showed higher initial scores for stress and anxiety than that of young people of the same age. Based on this finding, the authors suggest that even a small symptom reduction effect would be valid for these individuals. We could say that, although the students did not show severe symptoms and were not diagnosed with a mental illness, volunteering for a stress reduction program may reveal that, in the students’ perception, there was a need to improve their psychological health at some level. Therefore, any perceived improvement in this context would be beneficial.

In the follow-up assessments from 6 months to 1 year after the intervention, the meta-analyses showed a constancy of the small effect for the mindfulness outcome, of the moderate effect for the stress outcome, and of the large effect for the well-being/psychological health outcome. The effect constancy or small modification in the follow-up assessment may reinforce the hypothesis that there is no expectation of great improvement in a population that was not “ill,” besides confirming that the gains resulting from the training are important to the point of being sustained over time. Therefore, the perceived improvement in the well-being/psychological health may have become clearer to students after some time, as perceiving oneself better and becoming aware of their body, feelings, and well-being are the skills acquired with meditation training. Thus, right after the intervention, the training effects can be verified, but they are probably not consolidated to the point of all being consciously identified yet. The effects of the meditative practice are cumulative, becoming more evident over time as you exercise what has been learned. Besides, the students tend to better perceive the training benefits when exposed to stressful situations throughout the medical school program, when what was learned in training can be put into practice in challenging, concrete situations and students can assess the effects of the meditative practice as a stress management tool. It is important to note that assessing the perceived improvement in psychological distress and well-being symptoms is a complex issue, moreover, because the assessments were carried out by the very students—even considering the criteria of the instruments validated for such assessments, they can be subjective. It should also be considered that, in the psychological scenario, there are no measures able to define the behavior complexity in the real world and, for this reason, abstract construct measurements, such as psychological conditions, are arbitrary, being indirect assessments and lacking a context-based interpretation [55].

A clear example of what is discussed above is that people do not recognize their improvement just by the scores obtained in tests before and after an intervention [55], but they notice the improvement if they can focus more on their studies, have better sleep, and are more willing to perform tasks they considered difficult before the intervention. When results are assessed within the daily routine context, they can represent an important contribution to a given intervention, such as changes in the quality of life perceived and reported by the research participants [55]. Given this, some of the included studies openly asked students about the importance [49, 52], usefulness [48], or impact [51] of the program in reducing their stress. The feedback was positive: 67.1% of the students in the study by Damião Neto et al. [51] reported a positive difference in their lives, while 92% of the students in the study by Phang et al. [48] reported having benefited from the program and 100% of them would recommend the program to friends and relatives. In Astin [52], on a scale from 0 to 10, the mean response for the importance of the program was 9.3.

The results of our meta-analysis are supported by the literature that addresses the benefits of offering mindfulness training as a mental health promotion strategy, both for general university students [25] and students in health-related careers [17, 24, 28, 56, 57]. The following effects were found for general university students: stress reduction, including reduced levels of anxiety, depression, and cortisol response [17]; for students of health-related areas, the effects found were the following: stress [28, 57], anxiety, and depression reduction, improved mindfulness, mood, self-efficacy, and empathy [23, 24], and promotion of well-being and adaptive coping [58]. This study aimed to evaluate the effectiveness of the MBSR program, specifically in undergraduate medical students, a population identified as more stressed than peers from other courses. There are other research papers on mindfulness-based interventions in medical education. One of them suggests that medical schools should take advantage of evidence to guide the development of stress management programs [59]. Another study shows that, in 2014, two-thirds of medical schools in the USA already offered mindfulness programs as an option for the management of the students’ self-care and exhaustion [30]. Oro et al. [60] indicate that the use of mindfulness programs by physicians and medical students would be helpful to improve their self-care and their specific skills and competencies for professional practice. The systematic review by Daya and Hearn [24] assessed the effectiveness of mindfulness-based interventions in reducing depression, stress, burnout, and fatigue in medical students. Despite the methodological differences (different questions, outcomes, type of intervention, and inclusion criteria), the results agree with those obtained by our meta-analysis regarding the improvement in mental health and well-being right after training.

In our systematic review, there was a higher demand for MBSR programs by female students compared to male students. Although this finding may also be due to the predominance of female students in medical courses [61, 62], a previous systematic review that also included only medical students had a similar finding [24], as well as another systematic review that included students from several undergraduate courses [17]. Considering that the prevalence rates of anxiety and depression disorders and suicidal behavior are higher among female students [10, 17, 63], this population may have a higher demand for interventions with potential benefits for those who suffer from these conditions. In this aspect, Daya and Hearn [24] suggest that gender differences in emotional regulation may also indicate why women could benefit more from mindfulness practice, as women would be more likely to ruminate, while men externalize their distress through sports practices, for example. Since the mindfulness practice aims to reduce ruminative states, it would be a helpful technique in this context. The fact that most samples are composed of women students shows a greater vulnerability in this population and also shows that female students recognize the need for care and seek help to deal with difficulties, which may not occur with male students. This finding raises an important research question: is the proportion of men in studies on mental disorders low because these are more prevalent in women or because men have more difficulty recognizing states of vulnerability and asking for help? The studies included in our systematic review did not assess the proportion of women and men enrolled in medical schools.

When it comes to the moment, the medical course starts offering the training, the basis for the researchers’ choice is not known. It appears that those who offered the training in the first grades may had a more preventive intent, although students may have difficulties in clearly perceiving the stress issue and their own psychological distress. Going to university and attending a medical school alone are anxiogenic factors, with worsening stress levels upon starting clinical clerkships in a more traditional curriculum. In a more current curriculum, contact with patients and with the clinical environment already occurs from the beginning of the course, though the overload may be better evidenced by students in its final stage. In any case, it is important to intervene and promote health and quality of life at any stage of the medical school training, as one finding is undeniable: physicians are arriving at the specialization/residency program tired, and the prevalence of burnout syndrome is high among this group. In summary, the literature relates the acquisition of mindfulness to the promotion of a cognitive and emotional balance [28, 57, 60, 64]. Thus, by changing the focus from the disorder to health promotion, the results presented in our study can be encouraging for managers who plan to implement stress management and wellness program in their institutions.

About the results of primary studies, a hypothesis is that they could have been more robust if students had achieved the necessary discipline to fulfill the expected number of hours of practice at home in each study. This could make a difference in the results of all experiments, as the time spent in practice can influence the effectiveness [28]. Besides, mindfulness is not a passive intervention but needs constancy and regularity, requiring changes in habits and the practitioner engagement. For schools that intend to adopt this type of training, a way to encourage the practice continuity is to offer weekly, fortnightly, or even monthly practice sessions for those who have already undergone the face-to-face or online training. From another perspective, the various training format options can be positive, as mindfulness training could be adapted to each context and be integrated into the health professionals’ training programs [28].

Our study has some limitations. First, although the studies included in this review show a good response to mindfulness training by students, when looked at individually, their results cannot be generalized, as the context of each medical school can vary depending on the country and the curriculum structure, which affects the students’ behavior. Second, the convenience sample in studies that included only students that took the MBSR program is susceptible to a selection bias, as more distressed students would be more likely to apply for stress reduction interventions [54, 65]. Third, despite there was a growth in publications on this topic in the last two decades, few studies followed a randomized clinical trial design and, for this reason, most of them did not meet the inclusion criteria of this research. Fourth, it was not possible to perform meta-analyses regarding the anxiety, depression, empathy, and resilience outcomes due to the insufficiency of data for statistical calculation. Despite being contacted, the authors of these studies did not provide the requested data. Fifth, it was not possible to perform a meta-regression analysis, which is indicated in case of heterogeneity among the studies that make up a meta-analysis, because we did not have a minimum number of ten studies for each meta-analysis. Sixth, the specific MBSR technique with adaptations was chosen in an attempt to obtain a training with comparable results, which was possible only to some extent, as not everything can be compared. In this regard, the eight studies included showed differences in the MBSR training program, including the content and length of the intervention, and the student’s follow-up. However, the MBSR program teaches different meditation techniques (seated meditation, body sweeping, yoga movements, and walking meditation), including mindfulness exercises for daily activities and domestic practice. In this aspect, the students’ learning of the MBSR basic principles and applying these principles in their daily lives is more important than the way the intervention is taught and the MBSR program length. Thus, we opted to include studies that privileged training programs that adopted basic principles of the original program by Kabat-Zinn (1982) regardless of the duration of the intervention, even if they had different formats to meet the local realities. Furthermore, we had to choose to compare only the initial assessment and the time point immediately after the intervention. This frustrated the expectation of obtaining a better understanding of how long the results achieved by the training would last. Differences among the interventions did not enable an assessment of the effect of the number of training hours, number of meetings (face-to-face or online), or time of individualized practice on the outcomes.

A strength of this review is to have managed to collect randomized clinical trials with interventions based on the same technique, having the same foundations as those of the Kabat-Zinn “school” [31], known for its success in reducing stress, especially in patients with chronic illnesses and in non-clinical populations [27, 32, 36, 54]. Other strengths were the extensive search in important databases and the classification of all outcomes according to the GRADE tool.

The remaining challenge is to produce primary studies capable of monitoring students that have access to mindfulness training and practice throughout medical education and comparing them with students without mindfulness support. Qualitative research could provide more data regarding the participants’ general subjective perceptions about the training and its different variables: the relationship with the instructor or with the platform that makes the training available (audio, cell phone application); the group environment (face-to-face or online); possible exchanges of experiences in case there is a group; the importance of the topics covered in each stage of the training; the training of the meditative practice itself; and the difficulties to maintain the practice over time. With the social distancing due to the COVID-19 pandemic, training probably undergone more adaptations. A study suggestion is to compare the different types of offers: face-to-face or remote (online platform) group training and face-to-face or remote (online platform, recorded audio, mobile app) individual training.

## Conclusions

The results indicate that students who participated in mindfulness training noticed a reduction in stress/psychological distress symptoms, anxiety, and depression, reporting an improvement in the well-being/psychological health, mindfulness, resilience, and empathy constructs. The quality of evidence for the mindfulness outcome is high; for the stress and well-being/psychological health outcomes is moderate; for the anxiety, depression, and resilience outcomes, low; and for the empathy outcome, very low. However, the significant heterogeneity among studies should be considered when interpreting these findings. For practical application, the implementation of an MBSR program can improve the student’s well-being and, consequently, their academic performance, providing novice physicians with essential skills, such as being more reflective and empathetic in their clinical practice.

### Differences between the protocol and the systematic review performed

The modifications made were as follows:

1. *Title, question, and purpose of the review*

The title of the review was changed, expanding the focus from anxiety and depression symptoms to psychological distress (a term that includes anxiety, depression, and other disorders) and wellness promotion, which affected the question and the objective, which also has been expanded for the same reason.

2. *Outcomes*

The following outcomes were not found in the included studies: involvement in the study, self-compassion, self-regulation, self-efficacy, reflective practice, and academic performance.

3. *Type of studies included*

In an attempt to reduce the risk of bias related to the study design, we chose to include only randomized clinical trials.

4. *Schedule*

The expected completion date was April 2020, but due to a delay in registering the protocol and to personal difficulties generated by the COVID-19 pandemic, the schedule was reworked.

We consider that these modifications do not represent significant deviations from the protocol.

## Data Availability

The datasets supporting the conclusions of this article are included within the article.

## Abbreviations

MBSR: Mindfulness-based stress reduction
SRP: Stress Reduction Program
NREPP: The National Registry of Evidence-based Programs and Practices
PROSPERO: Prospective Registry of Systematic Reviews
PRISMA: Preferred Reporting Items for Systematic Reviews and Meta-Analyses
PICOS: Population, intervention, comparison, outcome
CD: Compact discs
RoB: Risk of Bias
GRADE: Grading of Recommendations Assessment, Development and Evaluation
SMD: Standardized mean difference
CI: Confidence intervals
SCL-90-R/GSI: The Hopkins Symptom Checklist 90 Revised
PSS-10: Perceived Stress Scale
GHQ: General Health Questionnaire
BSI/GSI: Brief Symptom Inventory
DASS: Depression, Anxiety, and Stress Scale
STAI-1 Form: The State-Trait Anxiety Inventory
MAAS: The Mindful Attention Awareness Scale
FFMQ: Five Facet Mindfulness Questionnaire
MHC-SF: Mental Health Continuum-Short Form
GWBS: General Well-Being Schedule
WHOQOL-BREF: World Health Organization Quality of Life
ECRS: Empathy Construct Rating Scale
JSPE: Jefferson Scale of Physician Empathy
RS-14: Resilience Scale
